# Loot

**Published:** 1874-02

**Authors:** 


### Loot.

### *Salivation.*

Dr. Ebstein reports in the Berliner Wochen Schrift, 1873, No. 25, experiments demonstrating the inhibitory action of atropine upon the chorda tympani nerve, practically applied in arresting salivation, which he believes to be due to irritation of this nerve. —*Practitioner.*

### *Treatment of Carcinoma Uteri.*

The *Practitioner* quotes from Sitz-Berder physic-med. Soc. zu Erlangen, 1873, R. Schroder's alleged successful treatment of this formidable malady by the application of the alcoholic solution of bromine (bromine one, alcohol five parts) to the seat of disease, after having removed all of the disease that was recognizable, (how, is not stated), and subsequently having applied the actual cautery deeply. Two cases of permanent cures are claimed.

### *Abortion.*

Dr. Pallen (St. Louis Med. and Surg. Journal, October, 1873), advises the induction of abortion to relieve excessive vomiting in pregnancy. He has performed it five times, under certain conditions, and *the patients all died.* Such an experience must be a pleasing retrospect in a physician's life.

### *Gastralgia.*

1. Ice bags to epigastrium for ten minutes, morning and evening. 2. Mustard poultice to same locality after ice. 3. Spoonful of shredded ice with sugar, morning and evening, every five minutes for an hour.—M. JONLIN, in La France Medicale.

### *Surgeon General Barnes.*

The French Academy of Medicine has honored itself by electing Surgeon General Barnes a corresponding member by an almost unanimous vote.—*Phila. Med. Times.*

### *Transactions of the American Medical Association.*

The Philadelphia Med. Times terms the "Transactions" the national disgrace. We will not quarrel with our neighbor of the "City of Brotherly Love" about a choice of epithets, but at the same time we must remind him that just as the great Sam. Johnson was compelled to admit that both Hell and Scotland had their uses, so he must admit that the "Transactions" have their uses too, for which we refer him to his own editorial of Nov. 8th, on "Professional Advertising," and to that of Dec. 13th, on a "Psychological Phenomenon," and exhort him to be charitable.

***Skin Grafting—Gonorrheal Orchitis.***

Dr. Alfred C. Girard, U. S. A., reports to the Philadelphia Med. Times the complete cure of an indolent ulcer of three inches diameter, within seven days, by the application of three skin grafts, the size of millet seeds, to its surface; a modification of the ulcerative process being perceptible in six hours.

The same surgeon recommends the application of "*elastic bands*," such as are used for stationery, in the place of the adhesive straps ordinarily used in the treatment of gonorrheal orchitis.

***Chorea.***

Dr. George S. Gerhard reports to the same journal the results of his treatment of thirty cases of chorea. The duration of the disease before treatment varied in different cases from two weeks to fifteen years. In ten cases, the right side alone was choreic; in five cases, the left; in seven, the entire body; in three, the entire body, but chiefly the right side; in two, the entire body, but chiefly the left side; in one, the head, shoulders and face; in one, the head and right arm; in one, both arms, head and neck were involved. Sixteen cases were cured; seven exhibited marked improvement; three were improved, and in four the result was unknown. Of the cures, three were treated with arsenic alone; one with arsenic and zinc; one with arsenic and iron; one with arsenic and purgatives; one with arsenic and chloral; one with arsenic and atropia; one with arsenic, chloral and the cold douche; one with arsenic, iron and bromide of potassium; two with arsenic, sulphate of zinc and the cold douche; one with arsenic, cimicifuga and the cold douche; one with sulphate of zinc; one with zinc and galvanism, and one with gymnastics. There were seven females and five males under ten years of age, and thirteen females and five males under twenty-one years of age. The paper is very interesting, and deserves careful study.

